# Supplementary figures and images for: Conducting non-pandemic research during the COVID-19 pandemic: A case study and documentary review
Source: PLOS Glob Public Health. 2025 Dec 26;5(12):e0005702. doi: 10.1371/journal.pgph.0005702 (PMC12742765; doi:10.1371/journal.pgph.0005702)

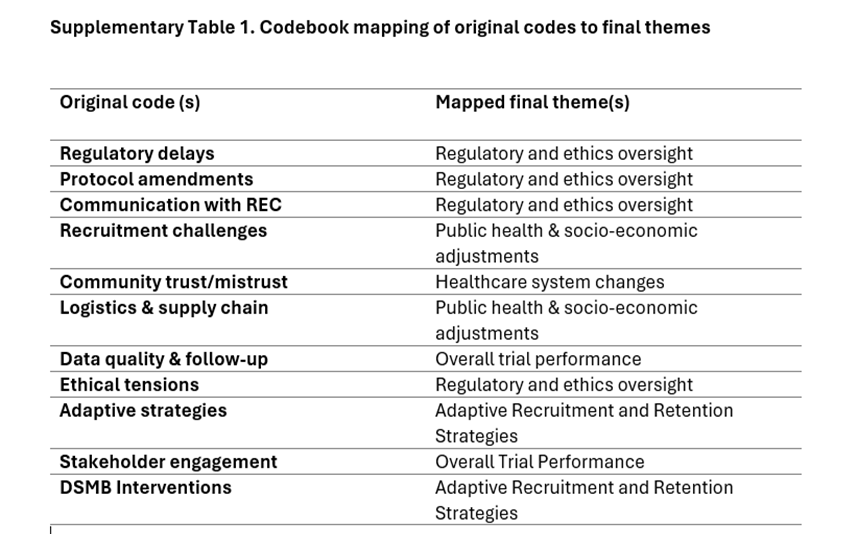

Supplement: S1 Table — (TIF) [file pgph.0005702.s001.tif]

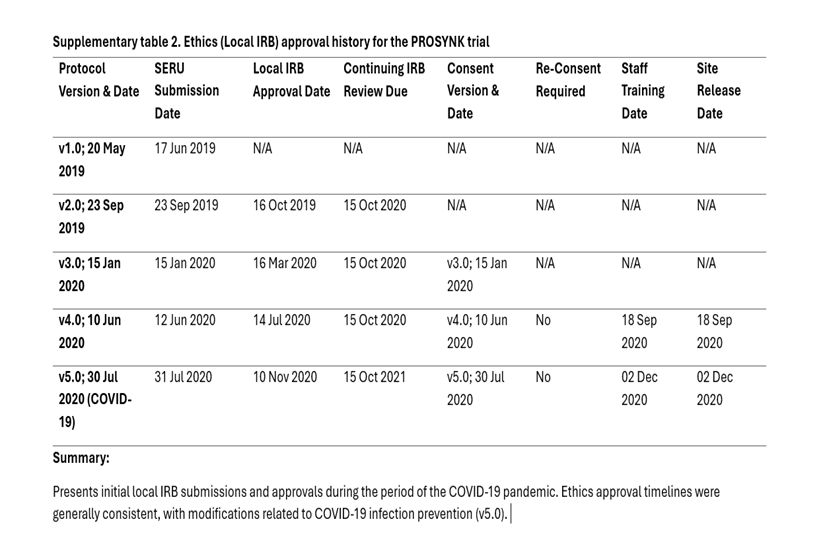

Supplement: S2 Table — (TIF) [file pgph.0005702.s002.tif]

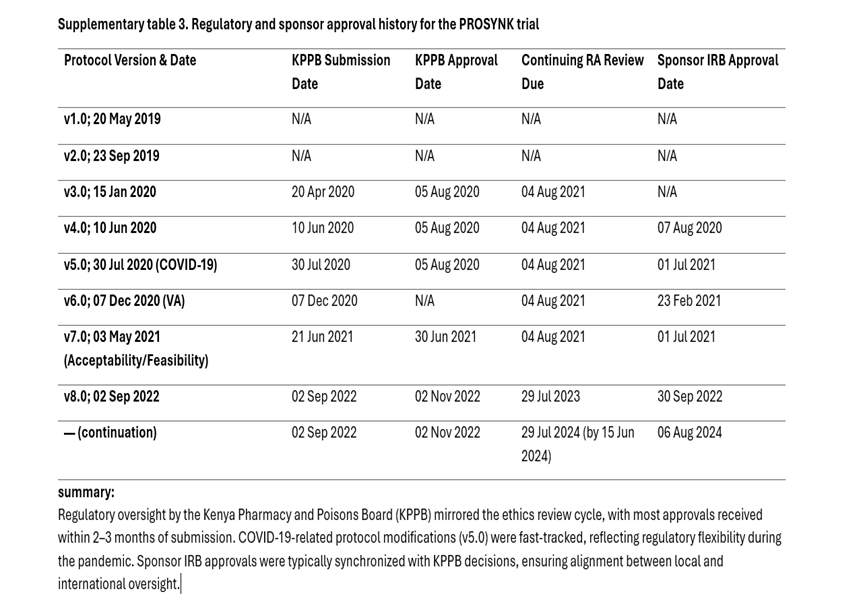

Supplement: S3 Table — (TIF) [file pgph.0005702.s003.tif]
