# Supplementary material for: Conducting non-pandemic research during the COVID-19 pandemic: A case study and documentary review
Source: PLOS Glob Public Health. 2025 Dec 26;5(12):e0005702. doi: 10.1371/journal.pgph.0005702 (PMC12742765; doi:10.1371/journal.pgph.0005702)
Supplement: Attachment — Submitted filename: Response to Reviewers.docx [file pgph.0005702.s005.docx]

Dear Editor and Reviewers

We wish to thank you for the detailed comments and feedback provided by such a thorough review. We are very grateful for the time you put into your review. We believe these have helped to improve our manuscript greatly. We look forward to hearing back from you and remain open to any further comments or feedback you may have.

We have responded to all of the items you raised. We added in red font our responses to all items. When we noted the lines in the manuscript where changes were made, these refer to the revised manuscript which has tracking still visible.

Thank you again for your feedback.

Journal Requirements:

1. Please amend your detailed Financial Disclosure statement. This is published with the article. It must therefore be completed in full sentences and contain the exact wording you wish to be published.

This has been done as requested.

i. State the initials, alongside each funding source, of each author to receive each grant.

Included in the Financial Disclosure statement.

ii. State what role the funders took in the study. If the funders had no role in your study, please state: “The funders had no role in study design, data collection and analysis, decision to publish, or preparation of the manuscript.”

The required wording was added to the Financial Disclosure statement: “The funders had no role in study design, data collection and analysis, decision to publish, or preparation of the manuscript.”

2. Please ensure that your Ethics Statement is available in its entirety at the beginning of your Methods section, under a subheading 'Ethics Statement'.

The Ethics Statement (formerly section 2.6) was moved to the start of the Methods section as section 2.1 and all subsequent section numbers adjusted.

3. Please provide separate figure files in .tif or .eps format.

For more information about figure files please see our guidelines:

[https://journals.plos.org/globalpublichealth/s/figures](https://urldefense.com/v3/__https:/journals.plos.org/globalpublichealth/s/figures__;!!KGKeukY!ybwQOTgHuIXMs2isrsCfHUUOlAZ97UuYHqDN-FDtpB9aQkxCMx9gc4ciYhHAHJSqGeDVsftitV9IAX-T_w$) 
[https://journals.plos.org/globalpublichealth/s/figures#loc-file-requirements](https://urldefense.com/v3/__https:/journals.plos.org/globalpublichealth/s/figures*loc-file-requirements__;Iw!!KGKeukY!ybwQOTgHuIXMs2isrsCfHUUOlAZ97UuYHqDN-FDtpB9aQkxCMx9gc4ciYhHAHJSqGeDVsftitV953S14Wg$)

All figures are now provided as *.tif files.

4. In the online submission form, you indicated that “Data supporting the findings of this study are available upon reasonable request. Interested parties should contact the corresponding author via email. Access will be granted following approval by all principal investigators involved in the original study.”.

All PLOS journals now require all data underlying the findings described in their manuscript to be freely available to other researchers, either 
1. In a public repository, 
2. Within the manuscript itself, or 
3. Uploaded as supplementary information.

This policy applies to all data except where public deposition would breach compliance with the protocol approved by your research ethics board. If your data cannot be made publicly available for ethical or legal reasons (e.g., public availability would compromise patient privacy), please explain your reasons by return email and your exemption request will be escalated to the editor for approval. Your exemption request will be handled independently and will not hold up the peer review process, but will need to be resolved should your manuscript be accepted for publication. One of the Editorial team will then be in touch if there are any issues.

We have sent a request for an exemption to the editor and journal. In this, we noted that we have made more of the details of our data available as supplementary files. These include the original codes developed as part of the thematic analysis (Supplementary Table 1.), a detailed history of the ethics review process (Supplementary Table 2.) and a similar history of the process through regulatory approval (Supplementary Table 1.). We have also added to the manuscript more details about these approval processes and the data analysis in response to reviewers’ specific requests.

However, much of our original data consists of direct correspondence between the PROSYNK team and the research ethics committee, Kenyan regulatory body and the study sponsor. In response to our request to make such documents publicly available, these bodies replied that these are confidential documents. These communications often include identifiable details of committee members and sensitive internal deliberations, which according to their established research governance standards are not meant for public release without formal institutional approval. We would therefore like to request an exemption to putting these documents into a public repository.

If the reviewer comments include a recommendation to cite specific previously published works, please review and evaluate these publications to determine whether they are relevant and should be cited. There is no requirement to cite these works unless the editor has indicated otherwise. 
The additional work mentioned by the reviewer was examined and was determined to be very relevant. It has been added to the list of references and the reference numbers adjusted.

Additional Editor Comments (if provided):


[Note: HTML markup is below. Please do not edit.]

Reviewers' comments:

Reviewer's Responses to Questions

**Comments to the Author**

1. Does this manuscript meet PLOS Global Public Health’s [publication criteria](https://urldefense.com/v3/__https:/track.editorialmanager.com/CL0/https:*2F*2Fjournals.plos.org*2Fglobalpublichealth*2Fs*2Fcriteria-for-publication/1/010f0198acb78451-7c80da85-65c3-4939-a8b1-ad0cdf50100e-000000/KXkIG5TxIPbwtgxKKW-0rrJs_G04M7id9W7gmGEEgNM=224__;JSUlJSU!!KGKeukY!ybwQOTgHuIXMs2isrsCfHUUOlAZ97UuYHqDN-FDtpB9aQkxCMx9gc4ciYhHAHJSqGeDVsftitV9PaFlU1A$)? Is the manuscript technically sound, and do the data support the conclusions? The manuscript must describe methodologically and ethically rigorous research with conclusions that are appropriately drawn based on the data presented.

Reviewer #1: Yes

Reviewer #2: Yes

2. Has the statistical analysis been performed appropriately and rigorously?

Reviewer #1: N/A

Reviewer #2: N/A

3. Have the authors made all data underlying the findings in their manuscript fully available (please refer to the Data Availability Statement at the start of the manuscript PDF file)?

The [PLOS Data policy](https://urldefense.com/v3/__https:/track.editorialmanager.com/CL0/https:*2F*2Fjournals.plos.org*2Fglobalpublichealth*2Fs*2Fdata-availability/1/010f0198acb78451-7c80da85-65c3-4939-a8b1-ad0cdf50100e-000000/hV5MhAOhPHg3qha6jSVSRpWaNVOWdtmKkwD-tC-a9v8=224__;JSUlJSU!!KGKeukY!ybwQOTgHuIXMs2isrsCfHUUOlAZ97UuYHqDN-FDtpB9aQkxCMx9gc4ciYhHAHJSqGeDVsftitV_ILiGmpA$) requires authors to make all data underlying the findings described in their manuscript fully available without restriction, with rare exception. The data should be provided as part of the manuscript or its supporting information, or deposited to a public repository. For example, in addition to summary statistics, the data points behind means, medians and variance measures should be available. If there are restrictions on publicly sharing data—e.g. participant privacy or use of data from a third party—those must be specified.

Reviewer #1: Yes

Reviewer #2: No

Addressed according to #4 above.

4. Is the manuscript presented in an intelligible fashion and written in standard English?

PLOS Global Public Health does not copyedit accepted manuscripts, so the language in submitted articles must be clear, correct, and unambiguous. Any typographical or grammatical errors should be corrected at revision, so please note any specific errors here.

Reviewer #1: Yes

Reviewer #2: Yes

5. Review Comments to the Author

Please use the space provided to explain your answers to the questions above. You may also include additional comments for the author, including concerns about dual publication, research ethics, or publication ethics. (Please upload your review as an attachment if it exceeds 20,000 characters)

Reviewer #1: This is a valuable manuscript that describes the implementation of a non-COVID-19 related trial during the COVID-19 pandemic in Kenya. The manuscript is mainly based on a review of trial-related documents, including meeting minutes, supplemented with review of the literature and other documents with information on wider pandemic response. The authors report on the research regulatory and operational challenges encountered by the trial including how the trial sponsors, investigators and oversight committees adapted the trial to ensure that it was implemented within the restrictions of the COVID-19 pandemic, and in scientifically valid and ethical manner.

Thank you for your encouraging comments.
It would be helpful for the authors to highlight the challenges encountered by the trial on account of being non-pandemic research conducted during a pandemic. This would be a unique addition to the extensive literature on ethical and practical research challenges during public health emergencies. Also, readers would benefit from additional information about the research team, methodology and context of the study. For example, reference is made of “significant delays” and “expedited approvals” without information on the expected timelines, other than study completion which was delayed by seven months.

We added some general additional material about this issue, starting at line 77. Further details regarding the response of the PROSYNK study were added starting at line 253 and 355.

Specific comments follow.
Title and Abstract
1. Given that the manuscript is manly based on document review, rather than other forms of qualitative research methods such as interviews, consider whether titling this as a “…description of conducting…” might be more appropriate than framing it as “The experience of conducting…” There is limited reporting of direct experience or views related to conducting the trial.

Thank you for this suggestion. We have changed the title accordingly (see line 1).
2. Lines 24 and 26-27: What are the “ethics and regulatory submissions” that were reviewed? Specifically, it’s unclear whether authors reviewed the comments/feedback that the trial received from the ethics review committee.

We clarified that our review included the feedback from the ethics and regulatory committees (line 28 and 121).
3. Line 27: Consider deleting “literature review” as one of the methods used to generate findings reported in this manuscript. This is because the approach to literature review is not described in the Methodology or outcomes presented in the Results.

We did not carry out a literature review. Line 27 stated that the case analysis included reference to “relevant peer-reviewed literature”.
4. Consider adding participant observation as one of the methods used to generate findings. It is indicated (Lines 102 -104) that the first author had direct experience of trial implementation and that this experience contributed (Lines 97-98) to findings reported in this manuscript.

We appreciate the reviewer’s suggestion here, but we do not think that our work included participant observation. No additional participant observations were conducted in the reported research, nor were the first author’s direct observations sought. The review was based on the documentary evidence. The first author’s experience was described in the interests of transparency and to point to the need to include additional document reviewers to address concerns about bias.
Introduction
5. Lines 54-56: The authors may consider expanding this section to clarify the key ethical and practical issues raised by the conduct of non-pandemic research during a pandemic. For instance, what is the ethical justification for conducting non-pandemic research during a pandemic.

Thank you for this suggestion. This section has been expanded (line 57ff.)
6. Throughout the manuscript, there is mention of the need for ethical research, but it’s unclear how ethics or ethical issues are conceptualized. The discussion seem to focus on apparent practical challenges, such as staff shortages, mobility restriction, with little evidence of ethics analysis.

Further discussion of ethical issues has been added as part of addressing Reviewer 2’s request for a theoretical framework.
7. Highlight that the trial protocol was submitted and received initial ethics approval before the COVID-19 pandemic (October 2019), and it’s only amendments and submission to regulators that were considered during the pandemic.

Thank you for highlighting how our original description did not clearly explain what happened. Changes have been introduced at sections starting on line 226. These hopefully make what happened here more clear.

Methods
8. Besides the first author, were any of the co-authors involved in the PROSYNK trial in any capacity? A fuller description of the research team, including roles authors in data collection and analysis, would help readers appreciate the insights, skills and experiences underlying the findings presented.

This was added with the section beginning as Line 98ff.

9. It would be great to describe the ethics and regulatory approval process for trials in Kenya during pandemic and non-pandemic periods, including expected turnaround times. This would provide a background for later discussions around regulatory/ethics delay and expedited approvals.

This has been added beginning with Line 123
10. Besides Document Access Log, were any software or platforms used for data management or analysis?

Since the number of included documents was not voluminous, themes were manually entered in Excel files. This has been noted on Line 188.
Results
11. There was a half-year delay between initial ethics approval and submission to regulatory authority as co-authors needed to amend protocol following change in the investigational product (Lines 149-150). Can the authors provided more information on why submitting this amendment took that long?

This has been elaborated on at Line 225ff.
12. How long did the trial investigators take to respond to comments/feedback from ethics committees and regulators? Overall timelines are given – from submission to approvals – but the contribution of trial investigators to review time is unclear.

This has been provided starting on Line 225.

### Reviewer #2:

This manuscript presents a retrospective documentary case analysis of the challenges faced by researchers when attempting to conduct a randomized controlled clinical trial (PROSYNK) investigating the effects of probiotics and synbiotics on infant gut health and systemic inflammation in a rural setting in western Kenya during the COVID-19 pandemic. The authors examine the ethical, operational, and logistical barriers encountered, as well as the adaptive strategies implemented to safeguard participant welfare and maintain study integrity during this public health emergency.


STRENGTHS
The research question is highly relevant and timely, addressing the underexplored topic of sustaining non-pandemic research during a global health crisis, particularly in low- and middle-income countries (LMICs). The manuscript is generally well written, logically structured, and free of superfluous content.
Thank you for your encouraging comments.
The methodological approach, a retrospective documentary case analysis, is well suited to the study’s objectives. The authors reviewed all available trial documents, including ethics submissions, meeting minutes, and progress reports, ensuring comprehensive coverage. Data analysis was conducted using dual coding, with a reported Cohen’s Kappa ≥ 0.6, which strengthens the reliability of findings. The use of a thematic framework analysis is appropriate and rigorously applied.
Thank you again for these comments.
The results are detailed and supported by quantitative trial performance indicators. Interpretation is solidly grounded in the data and enriched by comparisons with existing literature. The manuscript effectively emphasizes ethical responsiveness, community engagement, and participant protection, and it offers concrete, practical recommendations of clear value for sustaining clinical research during public health emergencies in LMICs.
Thank you.

MAJOR ISSUES
Two substantive issues must be addressed before the manuscript is suitable for publication:

a. Data sharing: In line with PLOS ONE’s recommendations, the authors should deposit the raw data in a publicly accessible repository. In this case, “raw data” should include the original taxonomy of themes from the first coding round and the final taxonomy after refinement.
We have sent a request for an exemption to the editor and journal. In this, we noted that we have made more of the details of our data available as supplementary files. These include the original codes developed as part of the thematic analysis (Supplementary Table 1.), a detailed history of the ethics review process (Supplementary Table 2.) and a similar history of the process through regulatory approval (Supplementary Table 1.). We have also added to the manuscript more details about these approval processes and the data analysis in response to reviewers’ specific requests.

However, much of our original data consists of direct correspondence between the PROSYNK team and the research ethics committee, Kenyan regulatory body and the study sponsor. In response to our request to make such documents publicly available, these bodies replied that these are confidential documents. These communications often include identifiable details of committee members and sensitive internal deliberations, which according to their established research governance standards are not meant for public release without formal institutional approval. We would therefore like to request an exemption to putting these documents into a public repository.

The original taxonomy of themes has been added as a supplementary file (suppl Table 1).

b. Absence of a developed theoretical framework: While the authors indicate an intention to ground their ethical discussion in principlism and public health ethics frameworks, this effort is underdeveloped and unconvincing. Beyond a brief, perfunctory reference in the Discussion (a single sentence, lines 257 - 259), there is no substantive engagement with principlism or any other explicit conceptual or theoretical framework to guide the analysis of adaptive strategies. Such absence limits the manuscript’s theoretical depth, resulting in a largely descriptive rather than analytically structured account. The authors should consider adopting a well-defined framework, such as resilience theory, implementation science, or a health systems framework, to organize complex ideas, connect findings to prior scholarship, and interpret results in context. A committed application of such a framework would require clearly justifying its selection, summarizing its core principles, and applying it consistently and rigorously to interpret findings, showing how results support, challenge, or expand upon the chosen theoretical perspective. This would situate the study within the broader academic conversation, establish a coherent theoretical basis, and yield more meaningful, generalizable conclusions.
Thank you for this important suggestion. We have added this material through the dual lens of principlism and resilience added to the introduction (line 77ff) and discussion sections (line 336ff).
SUGGESTIONS FOR IMPROVEMENT
Abstract
• Explicitly state that this is a case study of the PROSYNK Trial, which assessed the impact of probiotics and synbiotics on infant gut health and systemic inflammation in western Kenya.
This has been made more explicit (lines 24-25).
Study design
• Strengthen the justification for using a single case study and discuss how findings may be transferable to other settings.
This has been added starting at line 158.

Sampling
• Clarify how documents were included or excluded.
This has been added starting at line 172.

Data collection
• Describe how triangulation was achieved across document types.
This has been added starting at line 181.

Data quality and trustworthiness
• Briefly explain how potential biases arising from the authors’ direct involvement in the trial (lines 102 – 103) were mitigated.
This has been added starting at line 439.

Data analysis
• Provide more detail on the framework guiding coding and indicate any software used (NVIVO, ATLAS.ti). – No This was manually done

No software was used. A comment about this has been on line 188.

Results
• Discuss how specific adaptive measures, particularly the shift to remote follow-up in the control arm, could have affected trial validity.
A comment about this was added starting on line 388.

Tables
• Move Supplementary Table 2 (framework analysis of key themes) into the main text. Streamline it by merging the third column (“Key Questions and Responses”) with the second, as the separation is unnecessary.
This Table has been moved into the main text as Table 1 at line 193. The 2 columns have been merged as suggested.
Figures
• Consider simplifying figures and aligning them more closely with key findings.
We have simplified some of these and added the required captions.
• Evaluate whether Supplementary Figure 2 (Conceptual Framework) is necessary. In its current form it is overly complex, unreferenced in the main text, and potentially dispensable.

We agree with this comment and have deleted the figure.
Discussion
• Consider perhaps adding the following recent reference at the end of line 56, line 233, and line 277: Salamanca-Buentello F, Katz R, Silva DS, Upshur REG, Smith MJ (2024). Research ethics review during the COVID-19 pandemic: An international study. PLoS ONE, 19(4): e0292512. [https://doi.org/10.1371/journal.pone.0292512](https://urldefense.com/v3/__https:/doi.org/10.1371/journal.pone.0292512__;!!KGKeukY!ybwQOTgHuIXMs2isrsCfHUUOlAZ97UuYHqDN-FDtpB9aQkxCMx9gc4ciYhHAHJSqGeDVsftitV9HwD698w$)
Thank you for suggesting this article. It is very relevant and helpful and has been cited (reference 13) a few times.
Ethical, social, and cultural considerations
• Clarify how community input influenced adaptive strategies and how equity considerations were addressed in shifting to remote follow-up for the control arm.
This has been addressed starting at line 388.

Limitations
• Add a dedicated section acknowledging study limitations.
This has been added as Section 4.5 starting at line 427.

Contributions to knowledge
• Explicitly link recommendations to global guidance, particularly WHO and CIOMS guidelines on pandemic preparedness and research ethics during public health emergencies. The authors cite a key reference, the 2020 WHO report, but only in the Introduction.
This has been added at line 485.

Conclusion
• Address more explicitly the generalizability of findings beyond the PROSYNK context.
This has been added starting at line 481.

MINOR ISSUES
• Line 179: “Peer-reviewed studies and media reports …” Please provide the references for the sources mentioned.

This has been addressed and reference 11 added.

• Line 182: “…a news article dated 8 June 2021…” Again, please provide the appropriate reference.

This has been added at line 291 and is reference number 12.

• Line 246: Change “underscored” to “underscoring”, as the gerund is the correct form here.

This has been done.

Noted
• Line 299: “…Recruitment to target, high participant retention rates…” Add AND between “target” and “high” and remove the comma.

This has been done.

Noted
• Note that, in the PDF file received, Fig. 1 is out of place, as it appears after Figs. 2 and 3. This has been reconciled.

• If Supplementary Figure 2 is retained, correct “Participants safety and recruitment” to “Participants’ safety and recruitment”, as the apostrophe is needed to form the possessive.

This figure was removed.

SUMMARY
This is a valuable and well-executed case study that contributes to the literature on conducting non-pandemic research during public health emergencies in LMICs. Addressing the major concerns, particularly data sharing and strengthening the theoretical framing, will significantly enhance the paper’s analytical depth and policy relevance. With these revisions, the manuscript could offer important lessons for researchers, ethics review committees, and policymakers worldwide.

Thank you for these comments and all of your suggestions for improvements.

6. PLOS authors have the option to publish the peer review history of their article ([what does this mean?](https://urldefense.com/v3/__https:/track.editorialmanager.com/CL0/https:*2F*2Fjournals.plos.org*2Fglobalpublichealth*2Fs*2Feditorial-and-peer-review-process*23loc-peer-review-history/1/010f0198acb78451-7c80da85-65c3-4939-a8b1-ad0cdf50100e-000000/1SO47Ikoca4VF41MaXCZr_knUKlSJ4MJ8276g4wGV_Y=224__;JSUlJSUl!!KGKeukY!ybwQOTgHuIXMs2isrsCfHUUOlAZ97UuYHqDN-FDtpB9aQkxCMx9gc4ciYhHAHJSqGeDVsftitV_MLSR-LQ$)). If published, this will include your full peer review and any attached files.


**Do you want your identity to be public for this peer review?** If you choose “no”, your identity will remain anonymous but your review may still be made public.


For information about this choice, including consent withdrawal, please see our [Privacy Policy](https://urldefense.com/v3/__https:/track.editorialmanager.com/CL0/https:*2F*2Fwww.plos.org*2Fprivacy-policy/1/010f0198acb78451-7c80da85-65c3-4939-a8b1-ad0cdf50100e-000000/DTgxWaPqfOzCp7ke9lOq2t5R-VR4UaUeCuLITO2cNmE=224__;JSUl!!KGKeukY!ybwQOTgHuIXMs2isrsCfHUUOlAZ97UuYHqDN-FDtpB9aQkxCMx9gc4ciYhHAHJSqGeDVsftitV-I-4qxLA$).

Reviewer #1: No

Reviewer #2: No

[NOTE: If reviewer comments were submitted as an attachment file, they will be attached to this email and accessible via the submission site. Please log into your account, locate the manuscript record, and check for the action link "View Attachments". If this link does not appear, there are no attachment files.]

While revising your submission, please upload your figure files to the Preflight Analysis and Conversion Engine (PACE) digital diagnostic tool, [https://pacev2.apexcovantage.com/](https://urldefense.com/v3/__https:/pacev2.apexcovantage.com/__;!!KGKeukY!ybwQOTgHuIXMs2isrsCfHUUOlAZ97UuYHqDN-FDtpB9aQkxCMx9gc4ciYhHAHJSqGeDVsftitV9VJyAbtw$). PACE helps ensure that figures meet PLOS requirements. To use PACE, you must first register as a user. Registration is free. Then, login and navigate to the UPLOAD tab, where you will find detailed instructions on how to use the tool. If you encounter any issues or have any questions when using PACE, please email PLOS at [figures@plos.org](mailto:figures@plos.org). Please note that Supporting Information files do not need this step.

*In compliance with data protection regulations, you may request that we remove your personal registration details at any time.* [*(Remove my information/details)*](https://urldefense.com/v3/__https:/track.editorialmanager.com/CL0/https:*2F*2Fwww.editorialmanager.com*2Fpgph*2Flogin.asp*3Fa=r/1/010f0198acb78451-7c80da85-65c3-4939-a8b1-ad0cdf50100e-000000/ASczr8i5o8pXGHC5_gaxJZQuraApm2plCTeG5WdhnGE=224__;JSUlJSU!!KGKeukY!ybwQOTgHuIXMs2isrsCfHUUOlAZ97UuYHqDN-FDtpB9aQkxCMx9gc4ciYhHAHJSqGeDVsftitV9vRW5gQg$)*. Please contact the publication office if you have any questions.*
